# Increased risk of hearing loss associated with *MT-RNR1* gene mutations: a real-world investigation among Han Taiwanese Population

**DOI:** 10.1186/s12920-024-01921-8

**Published:** 2024-06-05

**Authors:** Hou-Kuang Chen, Yow-Wen Hsieh, Hsing-Yu Hsu, Ting-Yuan Liu, Yu-Ting Zhang, Chia-Der Lin, Fuu-Jen Tsai

**Affiliations:** 1 Department of Otorhinolaryngology-Head and Neck Surgery, China Medical University Hospital, Taichung, Taiwan; 2 Department of Pharmacy, China Medical University Hospital, Taichung, Taiwan; 3 Graduate Institute of Clinical Pharmacy, College of Medicine, National Taiwan University, Taipei, Taiwan; 4 Million-person precision medicine initiative, Department of Medical Research, China Medical University Hospital, Taichung, Taiwan; 5 Department of Medical Research, China Medical University Hospital, Taichung, Taiwan; 6 School of Chinese Medicine, College of Chinese Medicine, China Medical University, Taichung, Taiwan; 7 Division of Pediatric Genetics, Children’s Hospital of China Medical University, Taichung, Taiwan; 8 Department of Biotechnology and Bioinformatics, Asia University, Taichung, Taiwan; 9 School of Pharmacy, College of Pharmacy, China Medical University, Taichung, Taiwan

**Keywords:** Mitochondrial DNA, Mitochondrial 12S rRNA (*MT-RNR1*), Sensorineural hearing loss (SNHL)

## Abstract

**Background:**

Previous studies have implicated inherited mutations in mitochondrial DNA (mtDNA) in sensorineural hearing loss (SNHL). However, the definitive association between mitochondrial 12S rRNA (*MT-RNR1*) variants and hearing loss in the population has not been well established, particularly in Asia. The objective of this retrospective cohort study was to assess the association between *MT-RNR1* variants and the risk of SNHL in patients in Taiwan.

**Methods:**

The cohort included 306,068 participants from Taiwan between January 2003 and December 2020. Participants were classified based on genetic variants, particularly mitochondrial mutations (rs267606618, rs267606619, rs267606617). *MT-RNR1* variant cases were matched 1:10 with non-mutant patients by age, gender, and visit year, excluding those with pre-existing hearing loss. The primary endpoint was SNHL, identified using specific ICD-TM codes with a 90% positive predictive value. Medication exposure history was determined via self-report or electronic medical records in the hospital. Cox proportional hazard regression models were used to assess the association between *MT-RNR1* variants and hearing loss, adjusting for various covariates. Kaplan-Meier survival curves and log-rank tests compared hearing loss incidence between groups.

**Results:**

The mean age of the mtDNA variants group is 32.4 years, with a standard deviation of 19.2 years. The incidence density of hearing loss for the mutation group was 36.42 per 10,000 person-years (95% Confidence Interval [CI], 27.21–47.73), which was higher than the 23.77per 10,000 person-years (95% CI, 21.32–26.42) in the wild-type group (*p* = 0.0036). Additionally, diabetes mellitus was associated with an increased risk of developing SNHL in individuals with *MT-RNR1* variants (adjusted hazard ratio = 1.76 [95% CI, 1.00-3.09], *p* < 0.05).

**Conclusion:**

This study highlights the increased risk of hearing loss in patients carrying *MT-RNR1* variants, particularly those with diabetes mellitus. Future research that integrates genetic and clinical data is crucial for developing more precise interventions to monitor and treat hearing loss in this vulnerable population.

**Supplementary Information:**

The online version contains supplementary material available at 10.1186/s12920-024-01921-8.

## Introduction

Hearing impairment is one of the most common sensory deficits in human beings. It is estimated that nearly 5% of the global population suffers from hearing loss, affecting individuals across all age groups [1]. Hearing loss is more than just a sensory deficit; it fundamentally alters the way individuals connect with their surroundings and with others. From a medical perspective, the inability to hear can induce social deprivation or culminate in secondary health implications, such as depression, dementia [2, 3]. In addition to personal health issues, hearing loss has significant socioeconomically ramifications. Those with hearing impairment, their contribution in the workforce and productivity can decline [4]. Therefore, it is crucial to thoroughly understand the causes and processes of hearing loss. Such knowledge is important to crafting some prevention or treatment strategies, benefiting both individuals and the broader community.

Multiple factors can contribute to the development of sensorineural hearing loss (SNHL), including infectious or toxic causes, metabolic abnormalities, noise exposure, aging, diseases of ear, trauma, and genetic factors [5]. Genetic factors may account for up to half of cases of hereditary hearing impairment [6]. Among genetic causes of hearing loss, 30% are syndromic, featuring associated physical issues, whereas 70% are non-syndromic. In non-syndromic cases, autosomal recessive forms predominate, comprising 75–80% of cases, autosomal dominant forms make up about 20%, and X-linked, Y-linked, and mitochondrial inheritance collectively represent the remaining 5% [7]. Among the array of genes identified for non-syndromic hearing impairment to date, GJB2, SLC26A4, and the mitochondrial 12S rRNA (*MT-RNR1*) gene are much more prevalent than others across different populations [8].

Moreover, inherited mutations in mitochondrial DNA (mtDNA) are predominantly inherited in a maternal manner [8, 9]. The mtDNA contains 37 genes: 13 for cellular respiration proteins, 22 for tRNAs, and 2 (*MT-RNR1* and *MT-RNR2*) for rRNAs—*MT-RNR1* codes for 12S rRNA and *MT-RNR2* for 16 S rRNA [10]. The *MT-RNR1* gene transcribes a specific mitochondrial-derived peptide (MDP) that has been reported to have beneficial effects on metabolic stress conditions such as obesity, insulin resistance, and type 2 diabetes [11]. Mutations in the mitochondrial 12S rRNA gene (*MT-RNR1*) are linked to both aminoglycoside-induced and nonsyndromic hearing loss. Particularly, the homoplasmic mutations *1555 A>G* and *1494 C>T* in *MT-RNR1* are observed [12]. Given that cases of SNHL often involve a known history of aminoglycoside use, it is noteworthy that the m.1095 T>C mutation ranks as the third most prevalent among individuals of Chinese descent with SNHL who have been exposed to aminoglycosides [13, 14]. Despite these associations, there is considerable variability in the degree and onset of hearing loss, as well as different levels of penetrance, among families carrying these mutations [15]. The incidence of hearing impairment in patient with *MT-RNR1* gene mutation remains lacking in real world data.

Considering the limited data on the penetrance of mtDNA hearing loss in Asian populations, this retrospective cohort study utilized local data to investigate the incidence of hearing impairment in patients carrying *MT-RNR1* variants. These findings could have deep implications for clinical decision-making related to gene variants and healthcare practices within the local community. Hence, this study was performed to explore the association between *MT-RNR1* gene mutations and the risk of hearing impairment in the Taiwanese adult patients.

## Methods

### Data source

In this retrospective cohort study, we analyzed data from the China Medical University Hospital (CMUH) database from January 1, 2003, to December 31, 2020. The research was approved by the Ethics Review Board of China Medical University (CMUH111-REC1-176) and adhered to the (Strengthening the Reporting of Observational Studies in Epidemiology) STROBE reporting guidelines. Data analysis was conducted between February 1 and July 31, 2023. For an investigation into the sensorineural hearing loss (SNHL) risk associated with mitochondrial gene mutations, we employed an in-house genetic database, which stands as the most extensive and representative genetic repository in Taiwan. We gathered hearing loss data from the CMUH between January 2003 and December 2020, which offers a diverse range of health data, from clinical records to genetic details. Further information on CMUH database is available in prior research [16].

### Study design and participants

This cohort study was conducted in Taiwan, including a population of 306,068 participants from CMUH. We classified the participants based on genetic variants to evaluate their risk of hearing loss and associated health conditions. To ensure consistency in the study, we included individuals of all ages from Taiwan carrying mitochondrial gene mutations with high prevalence in Asia (rs267606618, rs267606619, and rs267606617) as variant cases. We also ensured the availability of DNA records, the presence of *MT-RNR1* variants, and the provision of informed consent. *MT-RNR1* variant cases and non-mutant patients were matched in a 1:10 ratio in terms of age, gender, and visit year. We also excluded individuals with pre-existing hearing loss to ensure that both groups were healthy at the time of study entry. The study addressed factors related to sensorineural hearing loss from January 2003 to December 2020. The primary endpoint was the identification of sensorineural hearing loss, determined through specific ICD-9-CM and ICD-10-CM codes with a 90% positive predictive value, supported by a requirement of three occurrences. (See supplementary Table S4, Additional file 4).

### Mitochondrial 12 S rRNA variants and deletion analysis

Our analysis utilized single nucleotide polymorphism (SNP) arrays from the Taiwan Precision Medicine Initiative (TPMI). The TPMv1 Array plate, based in Santa Clara, CA, USA, comprised 714,461 probesets. Genotype calling was facilitated by Affymetrix® Power Tools (APT). The first phase of the analysis involves sample quality control (QC), where we introduce a statistic called Dish-QC to assess the signal across non-polymorphic loci. We retain samples with a Dish-QC greater than 82%. In the second phase, we identify genotyped samples with a call rate (CR) lower than a default value of 97% and exclude samples that fail to meet the CR criteria. Additionally, the average CR must exceed 98.5%. For detailed information, please refer to the manufacturer’s instructions. The overall sample pass rate must be above 95%. Ultimately, we only retain probes marked as ‘Best recommended’ across all probes. For all individuals, we analyze the SNP missing rate, keeping only those individuals with a missing rate less than 10%. After quality checks, 686,432 variants and 347,954 individuals were retained. Genotypes for Affx-91,439,596 (m.1095 T>C, rs267606618), Affx-98,506,994 (m.1494 C> T, rs267606619), and Affx-89,025,706 (m.1555 A>G, rs267606617) were derived from the Axiom-TPM array.

### Stratification variables

Age was used as a primary stratification variable, grouped into 10-year intervals. We also stratified based on types of *MT-RNR1* variants, aminoglycosides, loop diuretics, and comorbidities to assess varying risks of hearing loss. Medication exposure history was determined either via self-report or by referencing the electronic medical records from CMUH in Taiwan. Exposure to aminoglycoside antibiotics or loop diuretics was defined as a patient having received at least one prescription (See supplementary Table S3, Additional file 3), whether oral or injectable, within the specified period. These records contained encrypted personal medical and demographic information.

### Statistical analysis

We examined the baseline characteristics and the prevalence of hearing loss among comparing groups with and without *MT-RNR1* variants by using the Chi-square test or Fisher’s exact test for categorical variables and the Student’s t-test or Mann-Whitney U test for continuous variables. Additionally, the normality was assessed using the Shapiro-Wilk test. Using patients’ medical histories, available in the CMUH database since 2003, we obtained information on comorbidities that may represent shared risk factors for hearing loss. Individuals with SNHL were diagnosed at least three outpatient visits or one inpatient record, and the first and last outpatient visits were at least 90 days apart. To avoid immortal time bias, the third diagnosis was designated as the index date. Comorbidities and medication usage preceding this date were identified as potential risk factors for SNHL. A comorbidity was confirmed and identified via specific ICD-9-CM and ICD-10-CM codes (See supplementary Table S4, Additional file 4). if there were at least three outpatient visits or one inpatient record with the associated code within a year. These consisted of all hospital inpatient and outpatient diagnoses of chronic kidney disease, diabetes mellitus, hypertension, otitis media, and hyperlipidemia. Secondly, we assessed the longitudinal association between *MT-RNR1* variants and the incidence of hearing loss by using Cox proportional hazard regression models with covariates including sex, age, the use of aminoglycoside or loop diuretics, and comorbidities (See supplementary Tables S3-S4, Additional file 3–4). Last, the Kaplan–Meier survival curve and log-rank test were applied to compare the incidence of hearing loss among groups with and without *MT-RNR1* variants. The 95% confidence intervals (CIs) of incidence of hearing loss were then calculated assuming the events followed Poisson distribution. The statistical significance level for all tests was set at a 2-sided level of 0.05. All statistical analyses were performed in Python, version 3.10 (Python Software Foundation).

## Results

**Fig. 1:**
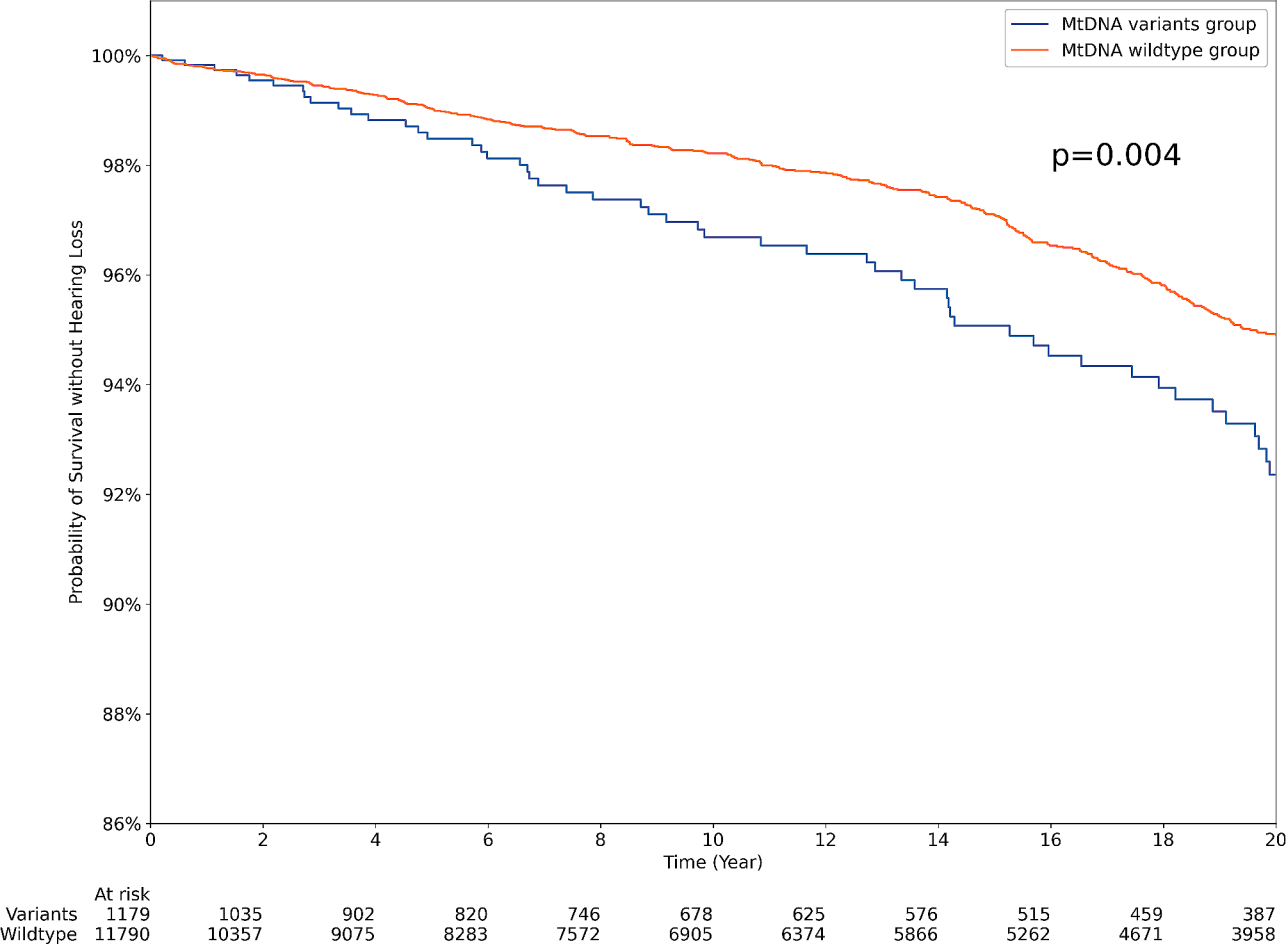
Kaplan–Meier estimates of hearing loss-free survival based on the presence of MT-RNR1 variants. The rate at which patients experienced Sensorineural Hearing Loss (SNHL) differed significantly depending on the identification of *MT-RNR1* variants (log rank test, *p* = 0.004)

In this cohort study, we analyzed data from 306,068 participants in Taiwan, utilizing the CMUH database. This was linked with the catastrophic illness database to confirm that the initial study population did not have hearing loss. We identified 1,179 individuals with MT-RNR1 variants, classifying them into the mtDNA variants group. The average age of the mtDNA variants group is 32.4 years, with a standard deviation of 19.2 years. Among this group, females comprise 46.6% (550 cases). Compared to the matched group with wild-type mtDNA, there were no significant differences in age, gender, or the incidence of comorbidities (Table 1). During the 20-year follow-up period, the subjects with mtDNA variants had a higher risk of developing SNHL compared with the non-mutation comparison group, with an incidence density of 36.42 per 10,000 person-years versus 23.77 per 10,000 person-years (Table 2). The adjusted hazard ratio (aHR) of mtDNA variants was 1.54 (95% CI, 1.15–2.06, *p* = 0.0036). In subgroup analysis stratified by age, a higher risk of developing SNHL was observed among individuals with mtDNA variants compared to their non-mutation counterparts in the age groups of 35–44 (*n* = 17, aHR = 2.52, 95% CI [1.47–4.32], *p* = 0.001) and 45–54 (*n* = 12, aHR = 1.9, 95% CI [1.03–3.54], *p* = 0.039). Figure 1 depicts Kaplan–Meier estimates of hearing loss-free survival curves, revealing a conspicuous probability of SNHL association between individuals mtDNA variants and those without mutation (log-rank test for trend: *p* = 0.004).

**Table 1:** Characteristics of *MT-RNR1* variants cases and patients with no mutation from CMUH

	MT-RNR1 variants				
	Yes		No		
	*N* = 1179		*N* = 11,790		
Variable	n	(%)	n	(%)	*P* value^*^
m. 1555 A>G only	607	51.5	-	-	
m. 1095 T>C only	573	48.6	-	-	
m. 1494 C>T only	14	1.2	-	-	
Age group (years)					0.99
<=20	299	25.4	3054	25.9	
> 20–40	502	42.6	4956	42	
> 40–60	283	24	2830	24	
> 60–80	85	7.2	850	7.2	
> 80	10	0.8	100	0.8	
**Age (years), mean (standard deviation)** ^**†**^	32.4	19.2	32.2	19.3	0.76
Sex					0.99
Female	550	46.6	5500	46.6	
Male	629	53.4	6290	53.4	
**Ever use of Aminoglycoside**	116	9.8	1217	10.3	0.638
**Ever use of Loop diuretics**	169	14.3	1607	13.6	0.531
**Cases of Hearing loss within the family**	77	6.5	689	5.8	0.374
**Comorbidities**					
Dyslipidemia	174	14.8	1736	14.7	0.947
Hypertension	259	22	2650	22.5	0.717
Diabetes mellitus	158	13.4	1816	15.4	0.075
Chronic kidney disease	77	6.5	768	6.5	0.99
Otitis media	30	2.5	316	2.7	0.856

^**†**^The mtDNA variant group had a mean of 32.4 years, with a standard deviation of 19.2 years. Non-mutation comparison group (wild-type mtDNA) had a mean of 32.2 years, with a standard deviation of 19.3 years

Table 3 shows the results of Cox proportional hazard regression models. Individuals with the m.1555 A>G variants exhibited a significant risk of developing SNHL (aHR = 1.97, 95% CI [1.35–2.86], *p* < 0.001), while no significant difference was found in those with m.1494 C>T variants (*p* = 0.443). Females with mtDNA variants have a higher risk of having SNHL compared to females without mutations. The aHR of mtDNA variants was 1.59 (95% CI, 1.06 − 2.38, *p* = 0.0262). The subjects with mtDNA variants who had diabetes mellitus had a higher risk of developing SNHL compared with the non-mutation counterparts, with an incidence density of 60.52 per 10,000 person-years versus 34.5 per 10,000 person- years. The aHR of mtDNA variants was 1.76 (95% CI, 1.00 − 3.09, *p* = 0.0499). No significant difference between two groups was observed in the risk of developing SNHL based on other comorbidities in our study. The association between the use of loop diuretic (*p* = 0.182) or aminoglycosides (*p* = 0.888) and risk of developing SNHL in individuals with mtDNA variants were not found to be significant.

**Table 2:** Overall and age-specific incidence densities of sensorineural hearing loss (SNHL) from CMUH

	MtDNA variants group			MtDNA wildtype group				
	Events, No (%)	Person-years	ID, per 10,000 person-years (95% CI)	Events, No (%)	person-years	ID, per 10,000 person-years (95% CI)	Adjusted HR† (95% CI)	*p*-value
**Overall**	52 (100.0)	14,278	36.42 (27.21–47.73)	343 (100.0)	144,305	23.77 (21.32–26.42)	1.54 (1.15–2.06)	0.0036
**Age, y**								
>=65	3 (5.8)	528	56.82 (11.73-165.13)	32 (9.3)	4318	74.1 (50.74-104.45)	0.81 (0.25–2.66)	0.731
55–64	7 (13.5)	1024	68.33 (27.52-140.29)	47 (13.7)	11,423	41.15 (30.25–54.68)	1.88 (0.84–4.19)	0.1233
45–54	12 (23.1)	1840	65.21 (33.74-113.62)	67 (19.5)	18,602	36.02 (27.92–45.72)	1.91 (1.03–3.54)	0.039
35–44	17 (32.7)	2554	66.55 (38.82-106.34)	64 (18.7)	25,328	25.27 (19.47–32.26)	2.52 (1.47–4.32)	0.001
< 35	13 (25.0)	8331	15.6 (8.31–26.67)	133 (38.8)	84,635	15.71 (13.16–18.62)	1.00 (0.56–1.77)	0.9965

*Abbreviations* aHR, adjusted hazard ratio; ID, incidence density

†The 95% CIs of incidence of hearing loss were then calculated assuming the events followed Poisson distribution

†Adjusted for sex, age, the use of aminoglycoside or loop diuretics and comorbidities

**Table 3:** Association between MtDNA variants and risk of sensorineural hearing loss (SNHL), by different variables

	Events, No (%)	person-years	ID, per 10,000 person-years (95% CI)	Events, No (%)	Person-years	ID, per 10,000 person-years (95% CI)	Adjusted HR† (95% CI)	*p*-value
**Overall**	52 (100.0)	14,278	36.42 (27.21–47.73)	343 (100.0)	144,305	23.77 (21.32–26.42)	1.54 (1.15–2.06)	0.0036
m. 1555 A>G	30 (57.7)	6,916	43.38 (29.28–61.86)	343 (100.0)	144,305	23.77 (21.32–26.42)	1.97 (1.35–2.86)	0.0004
m. 1095 T>C	22 (42.3)	7,434	29.59 (18.55–44.77)	343 (100.0)	144,305	23.77 (21.32–26.42)	1.18 (0.77–1.82)	0.4431
**Sex**								
Female	27 (51.9)	7,878	34.27 (22.6-49.82)	167 (48.7)	79,240	21.08 (18.0-24.52)	1.59 (1.06–2.38)	0.0262
male	25 (48.1)	6,400	39.07 (25.3-57.61)	176 (51.3)	65,065	27.05 (23.21–31.35)	1.50 (0.99–2.29)	0.0564
**Ever use of Aminoglycoside**	3 (5.8)	1,609	18.64 (3.85–54.37)	39 (11.4)	17,805	21.9 (15.58–29.93)	0.92 (0.28–2.98)	0.888
**Ever use of Loop diuretics**	8 (15.4)	2,295	34.86 (15.06–68.57)	47 (13.7)	22,229	21.14 (15.54–28.11)	1.67 (0.79–3.53)	0.1821
**Comorbidities**								
Dyslipidemia	14 (26.9)	2,857	49.0 (26.82–82.08)	101 (29.4)	27,079	37.3 (30.39–45.3)	1.45 (0.83–2.55)	0.1948
Hypertension	19 (36.5)	4,000	47.5 (28.62–74.07)	152 (44.3)	39,400	38.58 (32.7-45.21)	1.27 (0.79–2.04)	0.3308
Diabetes mellitus	14 (26.9)	2,313	60.52 (33.13-101.34)	93 (27.1)	26,958	34.5 (27.85–42.25)	1.76 (1.00-3.09)	0.0499
Chronic kidney disease	8 (15.4)	1,091	73.36 (31.72-144.03)	50 (14.6)	11,465	43.61 (32.39–57.46)	1.64 (0.77–3.46)	0.1964
Otitis media	8 (15.4)	402	198.86 (86.24-388.07)	72 (21.0)	4,081	176.42 (138.29-221.66)	0.96 (0.46-2.00)	0.9114

*Abbreviations* aHR, adjusted hazard ratio; ID, incidence density

†The 95% CIs of incidence of hearing loss were then calculated assuming the events followed Poisson distribution

†Adjusted for sex, age, the use of aminoglycoside or loop diuretics and comorbidities

## Discussion

To our knowledge, this is the largest retrospective cohort study conducted in Asia, focusing on mtDNA allele mutations with the aim of examining the risk of hearing loss in patients with mtDNA variants. In our study, we observed that patients carrying *MT-RNR1* variants exhibited a significantly lower rate of hearing preservation, particularly those with comorbidities, compared to a control group without these mutations.

Previous research has implicated inherited mtDNA mutations in both syndromic and nonsyndromic SNHL [12, 13, 15]. However, the extent to which post-lingual hearing loss and presbycusis are due to mtDNA mutations remains uncertain due to the potential for both inherent and acquired mutations [17]. In Taiwan, the common deafness-associated genes assessed, in order of prevalence included *GJB2*, *SLC26A4*, *OTOF*, *MYO15A*, and *MT-RNR1* [18]. The mitochondrial m.1555 A>G mutation, has a reported prevalence of 0.1% in newborn screenings and accounts for 3.2% of Taiwanese families with idiopathic SNHL [15, 19, 20]. While mtDNA mutation is present in 5–10% of post-lingual hearing loss cases, significant ethnic variations exist [21]. The prevalence of the m.1555 A>G mutation in Taiwanese idiopathic SNHL patients is comparable to other populations [21–24], and varied penetrance of SNHL across different haplogroups suggests the mtDNA background influences disease expression [25, 26]. Moreover, modifiers such as aminoglycosides antibiotics and genetic factors can modulate the phenotypic manifestation of mtDNA mutations, underscoring the complexity of their role in hearing loss [27].

Our results support the observation that populations with mitochondrial gene variants exhibit a higher differential risk of hearing loss. This aligns with other studies, but our findings provide additional data that was still lacking for the Taiwanese Han general population [12, 18]. These findings also underscore the necessity of genetic testing for tailored hearing loss interventions, including treatment, rehabilitation, prognosis, and family planning [28]. During the 20-year follow-up period of our enrolled cases, the incidence density of hearing loss in the mtDNA variants group was 36.42 per 10,000 person-years, compared to 23.77 per 10,000 person-years in the mtDNA wild-type group. According to the results of a review article [13], m.1555 A>G, m.1095 T>C, and m.1494 C>T are the most commonly documented mutations in Chinese subjects with sporadic aminoglycoside-induced and non-syndromic hearing loss. In our study, within the mtDNA mutation group, 607 patients (51.5%) had the m.1555 A>G mutation, 573 patients (48.6%) had the m.1095 T>C mutation, and only 14 patients (1.2%) had the m.1494 C>T mutation. The second most common mutation in the *MT-RNR1* in our study, m.1095 T>C, was also included in the analysis. However, based on findings from East Asian families in the current studies, it appears to lack pathogenic significance and has a low penetrance for deafness [29, 30].

We found that the presence of mtDNA pathogenic variants in the *MT-RNR1* is associated with a higher risk of hearing loss, especially in the younger population (The age of 35 to 54). This could be attributed to the interplay of environmental factors (diet, radiation, etc.) with inherited nuclear DNA and mtDNA susceptibility genes on a background of accumulating mtDNA mutations, further contributing to the progressive deterioration of mitochondrial function with age [27, 31], thereby diminishing the influence of the *MT-RNR1* gene [32]. So it’s possible that with increasing age, other genetic changes or pre-existing health conditions may influence the interpretation of our statistical impact of *MT-RNR1* on hearing loss. From previous studies, the penetrance for hearing loss and varies widely among reports and families, but the lower penetrance was noted after aminoglycoside exposure was excluded [30–33]. It was postulated that other nuclear modifier genes or nuclear background play a role in the phenotypic manifestation of SNHL [26, 34]. The increased risk of aminoglycoside-induced hearing loss is well-documented in previous literature [34]. The Clinical Pharmacogenetics Implementation Consortium Guideline offers treatment recommendations for aminoglycoside use based on *MT-RNR1* genotypes, suggesting alternatives unless the severity of infection outweighs the high risk of permanent hearing loss and no safe or effective alternatives are available [35]. However, the limited number of patients with *MT-RNR1* pathogenic mutations and a history of aminoglycoside or loop diuretic exposure led to no significant correlation with increased hearing loss susceptibility in real world. This could be attributed to the rarity of patients with mitochondrial gene mutations, the infrequent diagnosis of hearing loss following the use of aminoglycosides or loop diuretics, or the possibility that patients with a history of exposure are not recorded in the database, all of which could contribute to a reverse causal relationship. Therefore, routine genetic screening before aminoglycoside therapy is somewhat challenging in our current daily clinical practice. Besides, research on early detection and prevention of related comorbidities in young individuals with mtDNA variants is notably limited clinically.

The significant impact of comorbidities on the onset of hearing loss, especially in populations with *MT-RNR1* variants, is evident in our study. This impact was notably substantial in patients diagnosed with diabetes mellitus. Potential biological mechanisms of these genetic variants have been elucidated. Among these, the *MT-RNR1* gene contains the sequence for mitochondrial open-reading frame of the 12 S rRNA-c (MOTS-c), one of mitochondrial-derived peptides (MDPs), reported to possess metabolic protective properties in various models of metabolic dysfunction, such as diabetes mellitus [11, 36, 37]. In vivo studies have also confirmed the role of MOTS-c in enhancing insulin sensitivity and glucose homeostasis, with implications for the folate cycle, 5-aminoimidazole-4-carboxamide ribonucleotide (AICAR), and AMP-activated protein kinase (AMPK) signaling pathways [37].

On the other hand, in most mammalian cells, reactive oxygen species (ROS), primarily produced in the mitochondria, are reactive oxygen species also known as free radicals. The accumulation of ROS and the resulting oxidative damage have been shown to cause tissue dysfunction in various parts of the auditory system [38–40]. Considering that mtDNA is situated near the site of oxidative phosphorylation (OXPHOS) in the inner membrane of mitochondria, it is particularly vulnerable to ROS, leading to an increased susceptibility to mtDNA mutations. Additionally, substantial evidence indicates a strong correlation between the loss of mtDNA integrity, ROS accumulation, and various health conditions, including the aging process, neurodegenerative diseases [41, 42], and diabetes mellitus, characterized by insulin resistance and hyperglycemia [43, 44].

In the context of age-related hearing loss (ARHL), a majority of studies have affirmed that the build-up of mtDNA mutations over time results in mitochondrial dysfunction and subsequent degeneration of the cochlea and central auditory pathway, culminating in ARHL [45, 46]. While the impact of the three mtDNA mutation alleles explored in this study concerning this “vicious cycle” (referred to as the mitochondrial clock theory [47]) remains uncertain, we postulate that the onset of hearing loss is strongly associated with the accumulation of oxidative damage. Even in our study, we observed a significantly different risk of developing hearing loss in the mtDNA variant group after adjusting for age. This finding supports the hypothesis that mitochondrial gene translation, including processes like the oxidative phosphorylation (OXPHOS) system mentioned previously, may play a role in hearing loss as well [37].

In previous studies [48, 49], the association between SNHL, diabetes mellitus and cardiovascular disease has been established. However, the role of dyslipidemia and renal disease in SNHL remains limited or unclear [50, 51]. Our results also demonstrated a significant relationship between SNHL and diabetes, particularly in individuals with mtDNA variants. From a clinical perspective, there is a compelling rationale for actively monitoring the hearing of patients with *MT-RNR1* gene mutations, especially those with comorbid conditions like diabetes. In the era of precision medicine, it is recommended to screen individuals with mtDNA variants for hearing problem during their health exams, and multimorbidity should be considered when treating one of the comorbid condition in audiological care [52].

Establishing a causal link between *MT-RNR1* mutations and aminoglycoside-induced hearing loss is challenging due to limited data. Although unable to confirm a significant association between aminoglycoside exposure and hearing loss in individuals with pathogenic variants, our findings suggest that mitochondrial sequence variants may be more common in non-syndromic hearing loss cases than clinically acknowledged. Furthermore, our study fills gaps in the interpretation guidelines for mitochondrial variants, uncovering the impact of drug exposure on phenotypes not fully recognized in these guidelines, such as comorbidities like diabetes mellitus.

## Limitations and strengths

This study possesses several limitations. Firstly, defining the incidence of hearing loss events using the ICD-TM code from data could result in misclassification. However, the criteria, which requires at least three outpatient claims records with a gap exceeding 90 days between the first and last appointments for hearing impairment, should reduce the risk of such misclassification. Furthermore, given the cohort design, this misclassification likely results in a non-directional bias, potentially leading to an underestimation of the disease risk. Secondly, the diagnosis of hearing loss in our study does not differentiate between varying degrees of severity, which could potentially affect our risk assessments. Nevertheless, we posit that any auditory deficit, regardless of the degree of decibel loss, is considered hearing loss for the affected individual. Consequently, this lack of distinction may not significantly impact our study’s findings. However, it represents an area worthy of further exploration in future research, which should include a detailed analysis of the degrees of hearing loss and corresponding decibel levels. Due to constraints in accessing specific information from the database, we couldn’t account for all the environmental factors associated with hearing loss, such as noise exposure or lifestyle factors (e.g., smoking, and drinking). This may introduce residual confounding bias. Moreover, given the ethical imperative to safeguard participants, it is infeasible to conduct prospective trials determining if drug treatments might induce irreversible deafness in genetically predisposed patients. Lastly, considering causal inference and the timing of medication use, only a minority of carriers of the *MT-RNR1* variant in our dataset were exposed to aminoglycosides, potentially increasing the risk of a type II error. Future research is warranted to further discuss the implications for patients with diabetes mellitus who carry this variant and are treated with aminoglycosides, in order to validate our findings.

This study offers several merits. First, our study involved a retrospective cohort design with a 20-year follow-up period, exceeding the follow-up duration of several other studies. Second, to mitigate variability in disease severity between the two groups, which could influence study outcomes, we assessed the risk of hearing loss in individuals with gene variants, using a reference group of patients without such variants for comparison. Third, we provide additional evidence regarding the risks associated with mitochondrial gene variants, specifically concentrating on the pharmacogenetic loci m.1555 A>G and m.1095 T>C, to deliver further insights. Future research is required to validate our observations.

## Conclusion

In this 20-year cohort study, participants with *MT-RNR1* gene mutations exhibited a risk of hearing loss approximately 1.54 times higher than those without such mutations. This risk was particularly elevated in participants with diabetes mellitus. Our study provides valuable insights into the importance of preventing hearing loss events in adults with mitochondrial gene mutations, particularly in diabetic patients. Future genetic cohort studies related to hearing loss, better integrated with clinical characteristics, will enable more precise medical interventions, including targeted treatments and monitoring for hearing loss in patients with mitochondrial gene mutations.

### Electronic supplementary material

Below is the link to the electronic supplementary material.

Supplementary Material 1

Supplementary Material 2

Supplementary Material 3

Supplementary Material 4

## Data Availability

The datasets generated and/or analysed during the current study are available from the corresponding author on reasonable request. When we intend to use electronic medical record data from our hospital for research purposes, we must maintain data confidentiality. We have submitted a research proposal, obtain IRB approval, sign a data confidentiality agreement, a conflict of interest avoidance statement, and other documents. Additionally, we must obtain approval from the Hospital Medical Data Management Committee before granting permission to data users to operate the data (IRB number: CMUH111-REC1-176 in additional file). All patient data will ultimately be de-identified and unlinked to ensure confidentiality. Data users can only access the analysis results obtained after computation for publication, ensuring the confidentiality of the data.

## Abbreviations

AICAR: 5-aminoimidazole-4-carboxamide ribonucleotide
aHR: Adjusted hazard ratio
AMPK: AMP-activated protein kinase
ARHL: Age-related hearing loss
CIs: Confidence intervals
CR: Call rate
CMUH: China Medical University Hospital
ID: Incidence density
mtDNA: Mitochondrial DNA
MDP: Mitochondrial-derived peptide
*MT-RNR1*: Mitochondrial 12 S rRNA
MOTS-c: Mitochondrial open-reading frame of the 12S rRNA-c
OXPHOS: Oxidative phosphorylation
QC: Quality control
ROS: Reactive oxygen species
SNHL: Sensorineural hearing loss
SNP: Single nucleotide polymorphism
STROBE: Strengthening the Reporting of Observational Studies in Epidemiology
TPMIL: Taiwan Precision Medicine Initiative
