# Supplementary material for: Increased risk of hearing loss associated with MT-RNR1 gene mutations: a real-world investigation among Han Taiwanese Population
Source: BMC Med Genomics. 2024 Jun 5;17:155. doi: 10.1186/s12920-024-01921-8 (PMC11155076; doi:10.1186/s12920-024-01921-8)
Supplement: Supplementary file 1 — Supplementary Material 1 [file 12920_2024_1921_MOESM1_ESM.docx]

**Table S1. Overall and Age-Specific Incidence Densities of Hearing Loss from CMUH**

| **.** | **MtDNA variants group** | | | **MtDNA wildtype group** | | | |  |
| --- | --- | --- | --- | --- | --- | --- | --- | --- |
|  | **Events, No (%)** | **person-years** | **ID, per 10000 person-years (95% CI)** | **Events, No (%)** | **person-years** | **ID, per 10000 person-years (95% CI)** | **Adjusted HR† (95% CI)** | **p-value** |
| **Overall** | 63 (100.0) | 17085 | 36.88 (28.35-47.16) | 408 (100.0) | 172668 | 23.63 (21.39-26.03) | 1.58 (1.21-2.06)*** | 0.001*** |
| **Age, y** |  |  |  |  |  |  |  |  |
| >=65 | 5 (7.2) | 519 | 96.4 (31.4-223.5) | 33 (5.5) | 4502 | 73.3(50.5-102.8) | 1.51 (0.59-3.88) | 0.393 |
| 55-64 | 7 (7.3) | 1134 | 61.7 (24.9-126.8) | 61 (5.6) | 12393 | 49.2(37.7-63.2) | 1.26(0.57-2.76) | 0.566 |
| 45-54 | 16(11.2) | 2115 | 75.7(43.3-122.6) | 76(5.4) | 21544 | 35.3(27.8-44.1) | 2.28(1.33-3.92) | 0.003*** |
| 35-44 | 21(10.6) | 3221 | 65.2 (40.4-99.5) | 74(3.9) | 31389 | 23.6(18.5-29.6) | 2.75(1.69-4.47) | 0.001*** |
| <35 | 14 (2.1) | 10096 | 13.9(7.6-23.3) | 164 (2.4) | 102840 | 16.0(13.6-18.6) | 0.88(0.51-1.53) | 0.658 |
| †Adjusted for sex, age, the use of aminoglycoside or loop diuretics and comorbidities | | | | |  |  |  |  |
