# Supplementary material for: Increased risk of hearing loss associated with MT-RNR1 gene mutations: a real-world investigation among Han Taiwanese Population
Source: BMC Med Genomics. 2024 Jun 5;17:155. doi: 10.1186/s12920-024-01921-8 (PMC11155076; doi:10.1186/s12920-024-01921-8)
Supplement: Supplementary file 2 — Supplementary Material 2 [file 12920_2024_1921_MOESM2_ESM.docx]

**Table S2. Association Between MtDNA variants and Risk of Hearing loss, by different variables**

|  | **MtDNA variants group** | | | **MtDNA wildtype group** | | | | | | |  | |
| --- | --- | --- | --- | --- | --- | --- | --- | --- | --- | --- | --- | --- |
|  | **Events, No (%)** | **person-years** | **ID, per 10000 person-years (95% CI)** | **Events, No (%)** | **person-years** | **ID, per 10000 person-years (95% CI)** | | **Adjusted HR† (95% CI)** | | **p-value** | |  |
| **Overall** | 63 (100.0) | 17085 | 36.88 (28.35-47.16) | 408 (100.0) | 172668 | 23.63 (21.39-26.03) | | 1.58 (1.21-2.05)*** | | 0.001*** | |  |
| m. 1555A>G | 31 (49.2) | 8187 | 37.86 (25.74-53.70) | 408 (100.0) | 172668 | 23.63 (21.39-26.03) | | 1.71 (1.19-2.46) | | 0.004*** | |  |
| m. 1095T>C | 32 (50.8) | 8992 | 37.86 (25.74-53.70) | 408 (100.0) | 172668 | 23.63 (21.39-26.03) | | 1.44 (1.01-2.07) | | 0.046** | |  |
| **Sex** |  |  |  |  |  |  | |  | |  | |  |
| Female | 33 (52.4) | 9469 | 34.85 (24.00-48.91) | 210 (51.5) | 95093 | 22.08 (19.20-25.28) | | 1.54 (1.07-2.22) | | 0.023 | |  |
| male | 30 (47.6) | 7616 | 39.39 (26.59-56.19) | 198 (48.5) | 77576 | 25.52 (22.10-29.33) | | 1.64 (1.11-2.4) | | 0.012 | |  |
| **Ever use of Aminoglycoside** | 3 (4.8) | 1965 | 15.27  (3.15-44.56) | 49 (12.0) | 21837 | 22.44  (16.60-29.65) | | 0.69 (0.22-2.23) | | 0.54 | |  |
| **Ever use of Loop diuretics** | 8 (12.7) | 2850 | 28.07  (12.13-55.23) | 49 (12.0) | 27648 | 17.72  (13.11-23.42) | | 1.58 (0.75-3.33) | | 0.23 | |  |
| **Comorbidities** |  |  |  |  |  |  | |  | |  | |  |
| Dyslipidemia | 18 (28.6) | 3659 | 49.19  (29.18-77.63) | 115 (28.2) | 33986 | 33.84  (27.94-40.60) | | 1.75 (1.06-2.90) | | 0.029** | |  |
| Hypertension | 25 (39.7) | 5001 | 49.99  (32.38-73.71) | 170 (41.7) | 49694 | 34.21  (29.27-39.74) | | 1.56 (1.03-2.38) | | 0.038** | |  |
| Diabetes mellitus | 16 (25.4) | 2882 | 55.52  (31.77-90.01) | 103 (25.2) | 33573 | 30.68  (25.05-37.20) | | 1.86 (1.10-3.16) | | 0.025** | |  |
| Chronic kidney disease | 8 (12.7) | 1345 | 59.47  (25.71-116.84) | 55 (13.5) | 14353 | 38.32  (28.88-49.85) | | 1.49 (0.71-3.14) | | 0.281 | |  |
| Otitis media | 11 (17.5) | 478 | 229.96  (115.34-407.73) | 82 (20.1) | 4911 | 166.96  (133-206.82) | | 1.15 (0.61-2.16) | | 0.689 | |  |
| †Adjusted for sex, age, the use of aminoglycoside or loop diuretics and comorbidities | | | | |  | |  | |  | |  | |
