# Supplementary material for: Increased risk of hearing loss associated with MT-RNR1 gene mutations: a real-world investigation among Han Taiwanese Population
Source: BMC Med Genomics. 2024 Jun 5;17:155. doi: 10.1186/s12920-024-01921-8 (PMC11155076; doi:10.1186/s12920-024-01921-8)
Supplement: Supplementary file 3 — Supplementary Material 3 [file 12920_2024_1921_MOESM3_ESM.docx]

**Table S3. Definitions of medications and their ATC codes**

| *At least 1 medication claim* | |
| --- | --- |
| **Medication** | **ATC code** |
| Aminoglycosides |  |
| Tobramycin | J01GB01 |
| Gentamicin | J01GB03 |
| Kanamycin | J01GB04 |
| Neomycin | J01GB05 |
| Amikacin | J01GB06 |
| Loop diuretics | C03C |
