# Supplementary material for: Increased risk of hearing loss associated with MT-RNR1 gene mutations: a real-world investigation among Han Taiwanese Population
Source: BMC Med Genomics. 2024 Jun 5;17:155. doi: 10.1186/s12920-024-01921-8 (PMC11155076; doi:10.1186/s12920-024-01921-8)
Supplement: Supplementary file 4 — Supplementary Material 4 [file 12920_2024_1921_MOESM4_ESM.docx]

**Table S4. Definitions of diseases and their ICD-9/10 codes**

| *1 hospital admission or at least 3 outpatient visits* | | |
| --- | --- | --- |
| **Disease** | **ICD-9-CM** | **ICD-10-CM** |
| Hearing loss | 388, 389 | H90, H91 |
| Sensory hearing loss | 389.11, 389.12, 389.14, 389.18, 389.10, 389.8, 388.01, 388.2, 388.11, 389.8, 389.9 | H90.3, H90.4, H90.5, H91.0, H91.1, H91.2, H91.8, H91.9 |
| Dyslipidemia | 278 | E65, E66 |
| Hypertension | 401-405 | I10-I15 |
| Chronic kidney disease | 585, 586 | N18.4-N18.9, N19 |
| Diabetes mellitus | 250 | E08-E13 |
| Otitis media | 381, 382 | H65, H66, H67 |
